# Thrifty effect of subanesthetic‐dose S‐ketamine on postoperative opioids and its safety and analgesic effectiveness: A prospective, triple‐blind, randomized controlled, polycentric clinical trial

**DOI:** 10.1002/ibra.12104

**Published:** 2023-05-15

**Authors:** Jun Ding, Yun‐Mei Yu, Man Luo, Xu Fang, Dan‐Dan Tan, Han‐Rui Qin, Xue‐Feng Ren, Yong‐Guo Zhang, Tao Luo, Lei Chen, Wan‐Qiu Yu, Zhao‐Qiong Zhu

**Affiliations:** ^1^ Department of Anesthesiology Affiliated Hospital of Zunyi Medical University Zunyi Guizhou China; ^2^ Department of Anesthesiology People's Hospital of Anshuan City Anshun Guizhou China; ^3^ Department of Anesthesiology People's Hospital of Qiannan Qiannan Guizhou China; ^4^ Department of Anesthesiology The People's Hospital of Tongren Tongren Guizhou China; ^5^ Department of Anesthesiology Qian Xi Nan People's Hospital Qianxinan Guizhou China

**Keywords:** general anesthesia, opioid consumption, safety, S‐ketamine, subanesthetic‐dose

## Abstract

**Aim:**

To investigate the thrifty effects of subanesthetic‐dose S‐ketamine on postoperative opioids and its safety and analgesic efficacy.

**Methods:**

Four‐hundred and twenty patients were divided into the control group (CON group), the S‐ketamine 0.2 mg/kg group (ES0.2 group), and the S‐ketamine 0.3 mg/kg group (ES0.3 group) randomly. Major indicators include the Visual Analogue Scale (VAS), the times of compression with analgesic pumps after surgery, and analgesic drug consumption from anesthesia induction to 48 h after surgery. Minor records include vital signs, the use of vasoactive drugs, the Ramsay scores, the occurrence of adverse events including nervous system reaction, and the patient's satisfaction with anesthesia.

**Results:**

Compared with the CON group, VAS scores decreased in the ES0.2 and ES0.3 groups (*p* < 0.05). At 10 min after extubation, the VAS scores of the ES0.3 group were lower than that of the ES0.2 group (*p* < 0.05). The total number of compression with analgesic pumps of the ES0.3 group was lower than that of the CON group (*p* < 0.05). The opioid consumption after surgery of the ES0.3 group was lower than those of the CON group and the ES0.2 group (*p* < 0.05). The ES0.3 group's heart rate (HR) was faster but the use of vasoactive, drug consumption was less than the other two groups (*p* < 0.05). There were no significant differences in the incidence of postoperative adverse events and anesthetic satisfaction among the three groups.

**Conclusion:**

Subanesthetic‐dose S‐ketamine at 0.2–0.3 mg/kg especially the 0.3 mg/kg in general anesthesia induction can safely and effectively reduce postoperative pain and save postoperative opioid consumption.

## Graphical abstract

To investigate the thrifty effects of subanesthetic‐dose esketamine on postoperative opioids and its safety and analgesic efficacy. Four hundred and twenty patients were divided into the control group (CON group), the esketamine 0.2 mg/kg group (ES0.2 group), and the esketamine 0.3 mg/kg group (ES0.3 group) randomly. To analyze the Visual Analogue Scale (VAS) at 10 min after extubation, immediately returning to the ward, and 2, 24, and 48 h after surgery, the times of compression with analgesic pumps after surgery and analgesic drug consumption from anesthesia induction to 48 h after surgery mainly. Minor records include their vital signs, the use of vasoactive drugs, the Ramsay scores, the occurrence of adverse events including nervous system reaction, and patient's satisfaction with anesthesia.

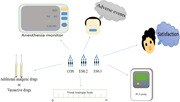

## 1 INTRODUCTION

Opioids are the most effective and widely used analgesic in clinical practice. However, administration of opioids would result in side effects including nausea, vomiting.^1^ It can also cause or worsen neurological symptom such as dizziness, agitation, delirium, headaches, and hyperalgesia.^2^, ^3^, ^4^ Reducing opioid consumption can decrease the risk of these adverse reactions in patients. Ketamine is an N‐methyl‐d‐aspartate (NMDA) receptor antagonist,^5^ and it is the only intravenous general anesthetic with analgesic properties. It initially found application in general anesthetic from 1970.^6^ Nevertheless, ketamine could excite the limbic system and lead to the separation of consciousness and sensation, thus triggering neurological adverse reactions including delusion, dissociation, or schizoid disorder.^7^, ^8^, ^9^ These reactions further limit ketamine's application in clinical practice. S‐ketamine is an isomer of ketamine and has excellent sedative and analgesic properties.^10^ It has a stronger affinity for NMDA receptors but fewer side effects than ketamine, providing a more comfortable experience for patients.^11^ Some clinical data have shown that S‐ketamine as a general anesthetic can be used safely and effectively in general anesthesia for many types of surgery at a dose of 0.5 mg/kg in clinical practice.^12^ For pediatric surgery for lower limb fracture, S‐ketamine at dose of 0.5 mg/kg combined with the nerve block has a perfect analgesic effect, fast anesthetic effect, and low incidence of pediatric agitation.^13^ This suggests that S‐ketamine does have a distinct advantage in the induction of general anesthesia. However, this dose of S‐ketamine has a high incidence of adverse reactions including neurological symptoms like dizziness and delirium.^14^, ^15^, ^16^ Finding a suitable dose of S‐ketamine, taking advantage of it, and avoiding adverse drug reactions at a high dose could be an interesting exploration. Therefore, two intermediate doses at 0.2 and 0.3 mg/kg were selected to observe.

## 2 MATERIALS AND METHODS

### 2.1 General data

This study was a prospective, triple‐blind, randomized controlled, polycentric clinical trial. We prepared the study medication beforehand and distributed it to the anesthetist using a computer‐generated randomization list. The syringes for the bolus injection were labeled with “Study Medication” and the randomization number of the patient in this study. The anesthetists were excluded from the evaluation of patients and data analysis. And the analyzer did not participate in grouping or administering anesthesia. The study was conducted in 15 hospitals including Affiliated Hospital of Zunyi Medical University. The time span is from June 2021 to December 2021. This study was approved by the Ethics Committee of the Affiliated Hospital of Zunyi Medical University and Hong Zheng who was the chairman of that committee. We got the number (KLL‐2020‐049) on 2021.04.30 and registered at the Chinese Clinical Trial Registry (no: ChiCTR2100046703). Patients (420) who underwent elective general anesthesia were randomly divided into three groups based on a ratio of 1:1:1. Nine patients still withdrew firmly after being reintroduced and comforted, and 411 cases were included in the statistics finally.

### 2.2 Inclusion criteria

(1) 18 years old ≤ age ≤ 64 years old; gender is not limited; (2) American Society of Anesthesiologists (ASA) I–II general anesthesia surgery patients; and (3) 18 kg/m^2^ < body mass index (BMI) < 30 kg/m^2^.

### 2.3 Exclusion criteria

(1) Contraindications to general anesthesia; (2) consumption of narcotic analgesic within 24 h or anesthesia drugs within 5 days and long‐term history of (continuous or intermittent) benzodiazepine sleeping pills and opioid analgesic; (3) history of severe cardiovascular disease; (4) history of hypertension with blood pressure not satisfactorily controlled by antihypertensive drugs; (5) history of head injury, possibly intracranial hypertension, cerebral aneurysm, cerebrovascular accident history, and central nervous system disease; history of elevated intraocular pressure (e.g., glaucoma) or punctured ophthalmic injury; (6) female with a history of drug and/or alcohol abuse within 2 years before screening; (7) gestational or lactating female; (8) history of hyperthyroidism; (9) history of psychiatric system diseases and drugs and cognitive dysfunction; and (10) history of participating in drug clinical trials as subjects in the past 3 months and patients who were not considered appropriate to participate in this trial by the investigators.

### 2.4 Withdrawal criteria

(1) Subjects actively withdraw the informed consent at any time; (2) occurrence of any clinical adverse events or other medical conditions that may cause more risks for the subjects; (3) the subjects were found to be unqualified after randomization; and (4) other reasons that result in discontinuation of the experiment.

### 2.5 Anesthetic methods

Patients routinely underwent to fast before surgery. The patients were admitted to the operating room on the day of surgery. Then, their peripheral venous access was routinely opened for intravenous fluids under monitored anesthesia care. Three groups of patients underwent routine general anesthesia with rapid sequence induction intravenously. For the CON group, the patients underwent conventional anesthesia induction (2.5 mg/kg of propofol, 0.3 μg/kg of sufentanil, and 0.9 mg/kg of rocuronium bromide). For the ES0.2 group, the patients underwent conventional anesthesia induction and were administered S‐ketamine (0.2 mg/kg). For the ES0.3 group, the patients underwent conventional anesthesia induction and were administered S‐ketamine (0.3 mg/kg). Routine intubation and mechanical ventilation were closely monitored intraoperatively to stabilize vital signs. A dose of sufentanil (10 μg) was administered before the end of surgery. The same patient‐controlled intravenous analgesia (PCIA) protocol was used after surgery. The formulation is listed below: 2 μg/kg of sufentanil, 8 mg of ondansetron, and 100 mL of diluted normal saline, followed by 2 mL/h of background infusion, 0.5 mL of single compression, and locking time for 15 min. Postoperative analgesics were nalbuphine, tramadol, dezocine, and sufentanil. The recorded data was taken as a baseline after the patients had been admitted to the operating room. The vital signs collected by the electrocardiogram (ECG) monitor were set at 0 min for the first time.

### 2.6 Observation indicators

Major indicators: (1) VAS scores at 10 min after extubation, immediately returning to the ward, and 2, 24, and 48 h after surgery (the assessment was carried out under the condition of patient consciousness); (2) analgesic drug consumption from anesthesia induction to 48 h after surgery; and (3) the times of compression with analgesic pumps within 2, 24, and 48 h after surgery.

Minor indicators: (1) Information about patients, anesthesia modes, and surgery; (2) records of vital signs upon admittance to the ward at rest, 5 min after induction, the moment of skin incision, 30 min after commencement of surgery, the moment of extubation, 10 min after extubation, the moment of returning to the ward, and 24 h, 48 h after surgery; (3) Ramsay sedation score at the moment of returning to the ward, 2, 24, and 48 h after surgery; (4) occurrence of adverse events within 2, 2–24, 24–48 h after surgery; and (5) anesthesia satisfaction (0–100 points).

### 2.7 Sample size calculation

When designing the study, the G‐Power 3.1 software was used for calculation, setting a error as 0.05 and (1−*β*) error as 0.95. The main observation selected the VAS score at 24 h after surgery, and the expected loss rate was designed to be 10%. We estimated that at least a total sample size of 377 patients would be required (126 in each group). To further reduce the bias, a population of 420 patients was considered to be included in this study.

### 2.8 Statistical methods

SPSS27.0 (International Business Machines [IBM]) was used to perform multiple comparisons between the three groups. The normal distribution data of the patients were expressed as mean ± standard deviations and were compared by analysis of variance. The nonnormal distribution data were expressed as median (minimum and maximum) and were compared by rank sum test. The counting data were expressed as frequencies and were compared by *χ* ^2^ test or Fisher's exact probability method. *p* < 0.05 was considered statistically significant.

## 3 RESULTS

### 3.1 General data about patients

**Figure 1:**
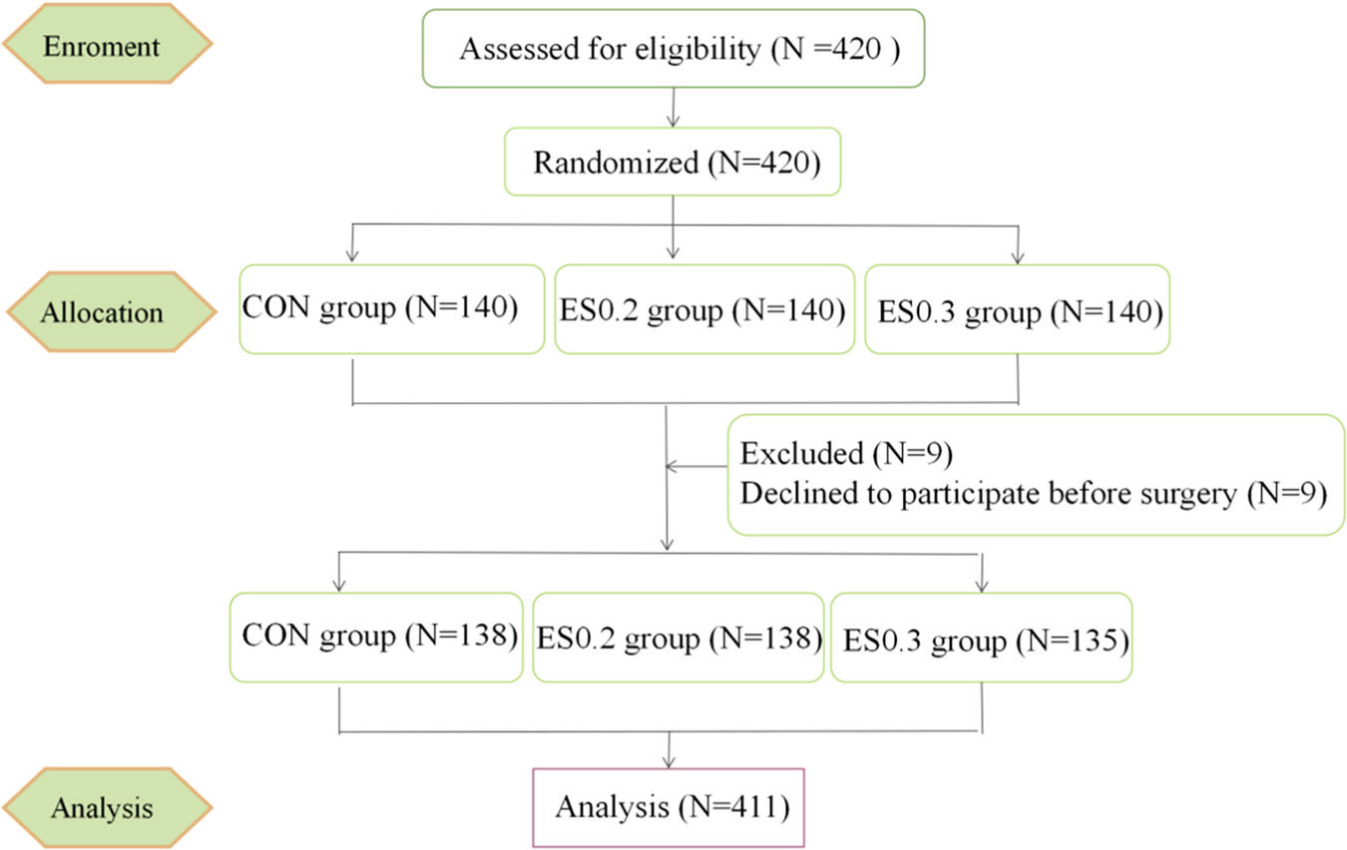
Flowchart of the study. CON, placebo; ES0.2, 0.2 mg/kg group; ES0.3, 0.3 mg/kg group. [Color figure can be viewed at wileyonlinelibrary.com]

Four hundred and twenty patients who underwent elective surgery were enrolled. Finally, 411 patients were included in the statistics: 138 cases in the CON group, 138 cases in the ES0.2 group, and 135 cases in the ES0.3 group (Figure 1). There was no significant difference in general data and surgical conditions among the three groups (*p* > 0.05), and the baseline of the three groups was consistent and comparable (Table 1).

**Table 1:** Comparison of the basic data between groups.

	CON (*N* = 138)	ES0.2 (*N* = 138)	ES0.3 (*N* = 135)	*F* value	*p* Value
Age (years)	39.4 ± 10.7	41.9 ± 11.5	40.5 ± 10.5	1.862	0.157
Height (cm)	157.8 ± 6.0	157.8 ± 6.8	157.6 ± 6.0	0.024	0.976
Weight (kg)	57.2 ± 7.7	58.7 ± 8.8	57.4 ± 8.2	1.395	0.249
BMI (kg/m^2^)	23.0 ± 2.7	23.5 ± 2.8	23.1 ± 2.9	1.146	0.319
Liquid intake and output (mL)					
Infusion	1130.4 ± 395.2	1221.2 ± 440.5	1210.0 ± 445.6	1.923	0.148
Bleeding	63.7 ± 98.3	67.5 ± 133.1	64.0 ± 56.7	2.113	0.122
Urine output	202.8 ± 170.2	215.2 ± 214.2	209.0 ± 200.5	1.968	0.869
Time (min)					
Anesthesia	120.2 ± 49.4	131.3 ± 62.5	119.5 ± 53.3	1.968	0.141
Surgery	96.8 ± 47.5	106.9 ± 58.9	95.0 ± 51.0	2.007	0.136
Emergence	22.4 ± 16.2	23.9 ± 18.8	21.5 ± 13.1	0.758	0.469
Stay in PACU	49.1 ± 17.4	53.8 ± 22.9	48.0 ± 13.1	2.442	0.088

*Note*: Data are presented as the mean ± standard deviation.

Abbreviations: CON, placebo; ES0.2, 0.2 mg/kg group; ES0.3, 0.3 mg/kg group; PACU, postanesthesia care unit.

### 3.2 Major indicators

#### 3.2.1 Postoperative pain assessment

Different surgical methods will affect the degree of pain. To reduce the interference of surgical methods on VAS scores, we divided them into open operation and nonopen operation. Then, comparisons among groups were conducted. In the CON group, ES0.2 group, and ES0.3 group, the proportion of open operation was 8.0%, 8.0%, and 6.7% separately.

##### VAS scores at each time point in patients undergoing nonopen surgery

**Figure 2:**
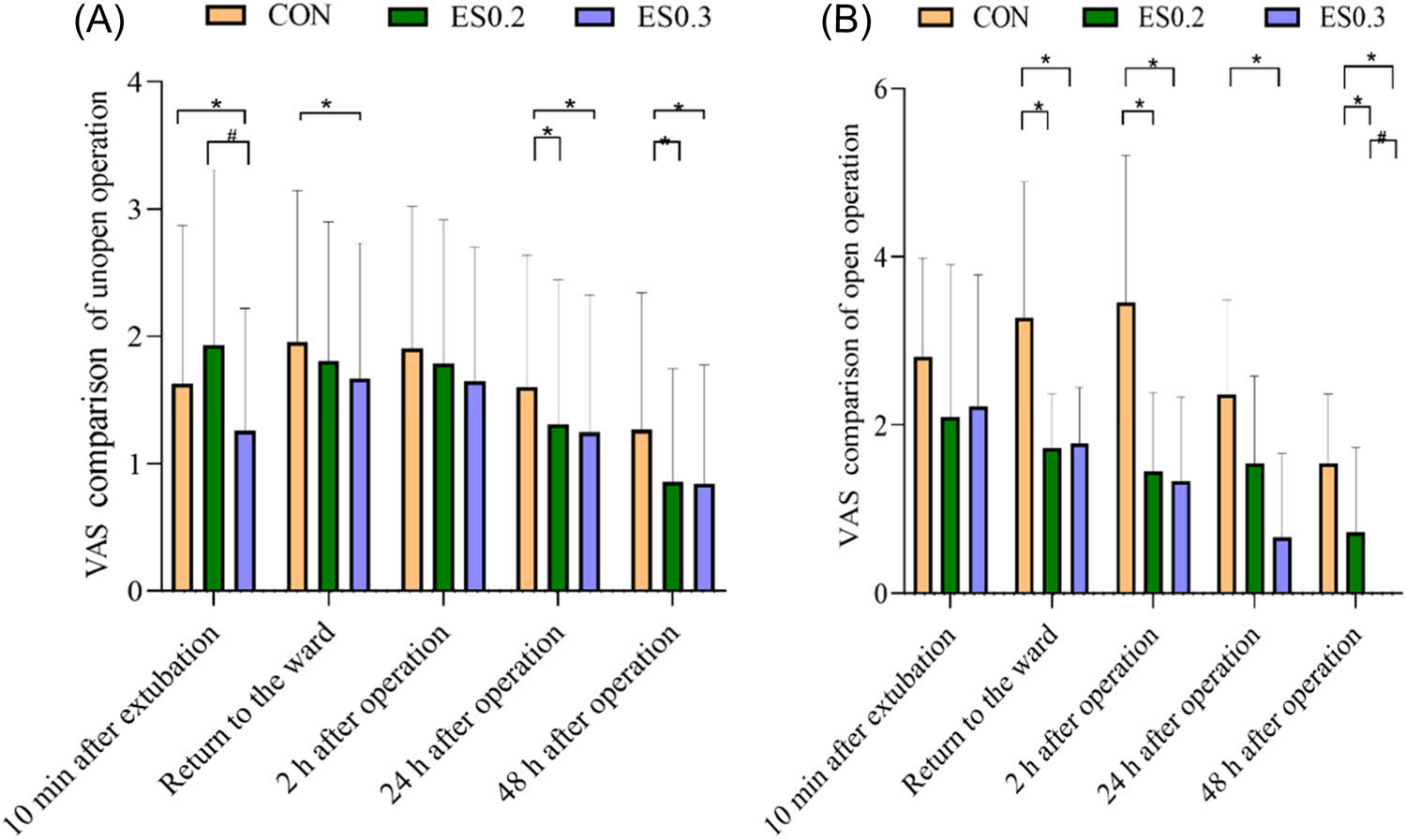
(A) VAS scores at each time point in patients undergoing nonopen surgery. (B) VAS scores at each time point in patients undergoing open surgery. The data are expressed as mean ± standard deviation. *In comparison with the Con group, *p* < 0.05; ^#^in comparison with the ES0.3 group *p* < 0.05. CON, placebo; ES0.2, 0.2 mg/kg group; ES0.3, 0.3 mg/kg group. [Color figure can be viewed at wileyonlinelibrary.com]

Compared with the CON group, the VAS scores were lower in the ES0.2 group at 24 and 48 h postoperatively (*p* < 0.05). The VAS scores were smaller in the ES0.3 group (*p* < 0.05) at 10 min after extubation, returning to the ward, 24 and 48 h after surgery. Compared with the ES0.3 group, the VAS score of the ES0.2 group was higher at 10 min after extubation (*p* < 0.05) (Figure 2A).

##### VAS scores at each time point in patients undergoing open surgery

Eleven patients from the CON group, 11 from the ES0.2 group, and 9 from the ES0.3 group underwent open surgery. The VAS scores of the ES0.2 group were lower than those of the CON group at the moment of returning to the ward, 2 and 48 h after surgery. The VAS scores of the ES0.3 group were lower than those of the CON group at the moment of returning to the ward, 2, 24, and 48 h after surgery. Especially 48 h after surgery, the VAS scores of patients from the ES0.3 group were all 0. Compared with the ES0.3 group, the VAS score of the ES0.2 group was higher at 48 h after operation (*p* < 0.05) (Figure 2B).

#### 3.2.2 Compression with analgesic pumps

Compared with the Con group, the total times of using pumps per person in the ES0.3 group were less (*p* < 0.05). In the three groups, there was no significant difference in the times of using pumps in the three groups within 24 h after surgery (*p* > 0.05), and there were fewer pumps per person in the ES0.2 group and ES0.3 group at 24 to 48 h after surgery (*p* < 0.05) (Table 2).

**Table 2:** Using compression with analgesic pumps.

	CON (*N* = 138)	ES0.2 (*N* = 138)	ES0.3 (*N* = 135)	*Z* value	*p* value
Total (times)	1.7 (0.22)	1.3 (0.15)	0.9 (0.10)^a^	3.542	0.030
0–2 h (times)	0.3 (0.4)	0.2 (0.3)	0.2 (0.2)	1.571	0.209
2–24 h (times)	0.9 (0.12)	0.9 (0.12)	0.6 (0.10)	1.036	0.356
24–48 h (times)	0.5 (0.10)	0.2 (0.5)^a^	0.1 (0.2)^a^	8.287	0.016

*Note*: Data are presented as median (minimum, maximal).

Abbreviations: CON, placebo; ES0.2, 0.2 mg/kg group; ES0.3, 0.3 mg/kg group.

^a^ In comparison with the Con group, *p* < 0.05.

#### 3.2.3 Opioid consumption

The opioids used during the operation were sufentanil and remifentanil. There was no significant difference among the three groups in their consumption (*p* > 0.05). When different postoperative opiate dosages were converted to morphine dosages based on the potency of the drug, the ES0.3 group consumed less opiates than the CON and ES0.2 group (*p* < 0.05) (Table 3).

**Table 3:** Consumption of opioids.

	CON (*N* = 138)	ES0.2 (*N* = 138)	ES0.3 (*N* = 135)	*F/Z* value	*p* Value
Sufentanil (μg)	17.0 ± 2.7	17.4 ± 2.5	17.1 ± 2.6	5.525	0.203
Remifentanil (mg)	0.9 ± 0.5	0.9 ± 0.6	0.9 ± 0.5	6.372	0.672
Morphine (mg)^a^	0 (0.10)	0 (0.10)^b^	0 (0.11)^c^	42.643	<0.05

*Note*: The data are expressed as mean ± standard deviation.

Abbreviations: CON, placebo; ES0.2, 0.2 mg/kg group; ES0.3, 0.3 mg/kg group.

^a^ Nonnormal distribution data are presented as median (minimum, maximal).

^b^ In comparison with the Con group, *p* < 0.05.

^c^ In comparison with the ES0.3 group, *p* < 0.05.

### 3.3 Minor indicators

#### 3.3.1 Changes in vital signs

**Figure 3:**
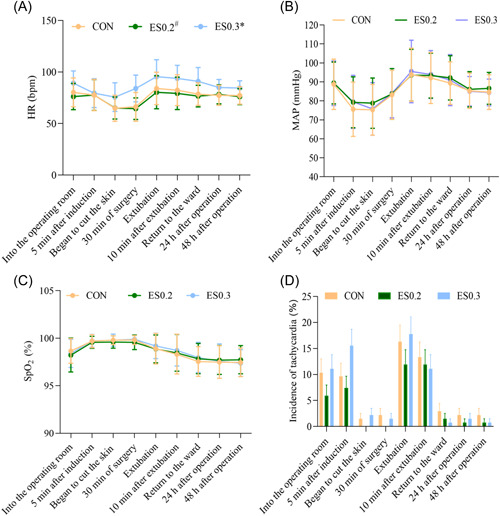
(A) Changes in the heart rate. (B) Changes in mean arterial pressure. (C) Changes in oxygen saturation. (D) Incidence of tachycardia (HR > 100 times/min). The data are expressed as mean ± standard deviation. *In comparison with the Con group, *p* < 0.05; ^#^in comparison with the ES0.3 group, *p* < 0.05. CON, placebo; ES0.2, 0.2 mg/kg group; ES0.3, 0.3 mg/kg group. [Color figure can be viewed at wileyonlinelibrary.com]

Except for 5 min after induction, the heart rate (HR) of the ES0.3 group was faster than that of the CON group and the ES0.2 group at other time points, and the average HR was 10.9 beats/min faster (*p* < 0.05) (Figure 3A). There was no significant difference in the incidence of tachycardia, mean arterial pressure (MAP), and oxygen saturation (SpO_2_) between the three groups at each time point (*p* > 0.05) (Figure 3B–D).

#### 3.3.2 Vasoactive drugs

The ES0.3 group's consumption rate of vasoactive drugs was fewer than that in the CON group and the ES0.2 group (*p* < 0.05) (Table 4).

**Table 4:** Consumption rate of vasoactive drugs.

	CON (*N* = 138)	ES0.2 (*N* = 138)	ES0.3 (*N* = 135)	*X* ^2^ value	*p* value
Consumption rate (%)	31.9	38.4^a^	21.0^b^	9.560	0.008

*Note*: The data are expressed as the percentage.

Abbreviations: CON, placebo; ES0.2, 0.2 mg/kg group; ES0.3, 0.3 mg/kg group.

^a^ In comparison with the ES0.3 group, *p* < 0.05.

^b^ In comparison with the Con group, *p* < 0.05.

#### 3.3.3 Respiratory circulation

**Figure 4:**
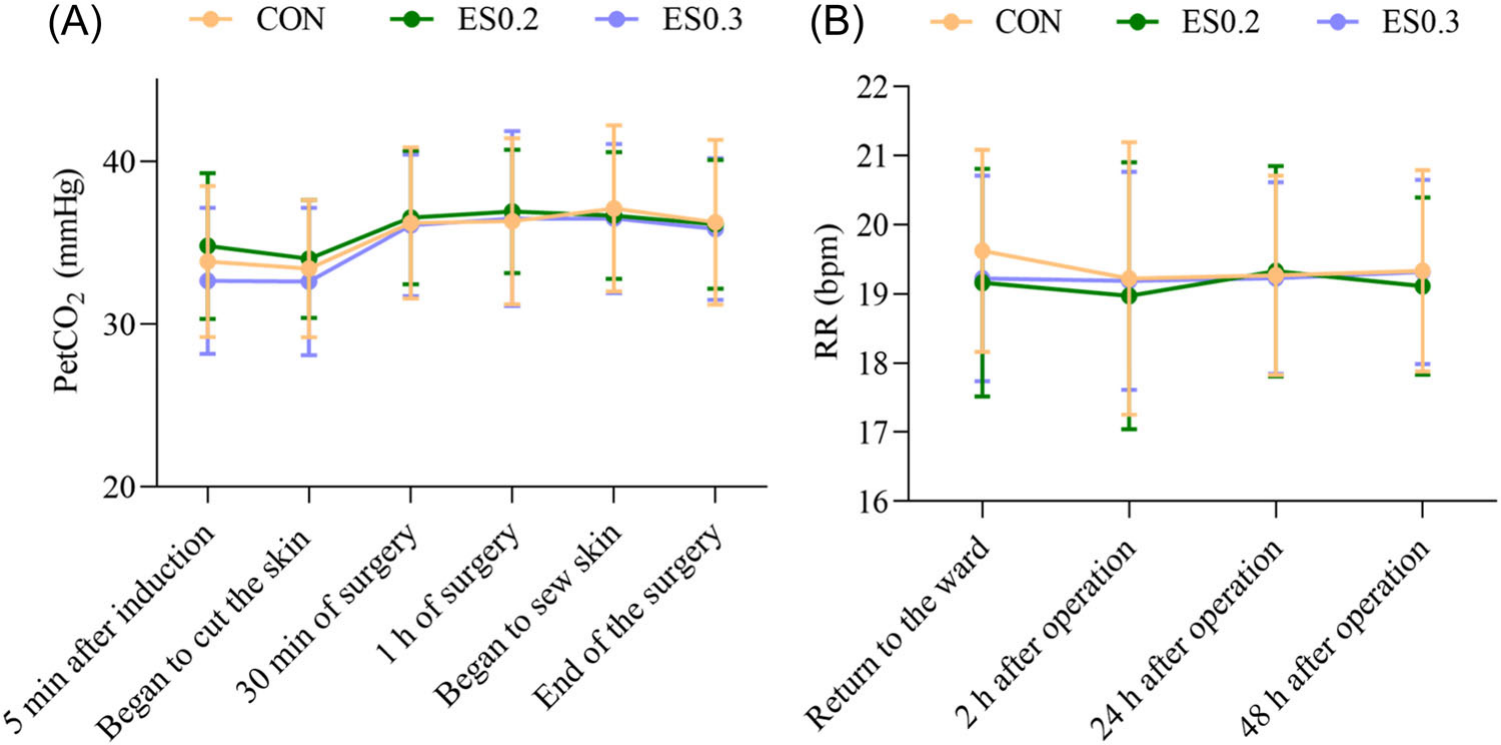
(A) Intraoperative partial pressure of end‐tidal carbon dioxide. (B) Postoperative respiratory frequency. The data are expressed as mean ± standard deviation. CON, placebo; ES0.2, 0.2 mg/kg group; ES0.3, 0.3 mg/kg group. [Color figure can be viewed at wileyonlinelibrary.com]

The partial pressure of end‐tidal carbon dioxide and respiratory frequency was stable in the three groups. There was no significant difference among the groups (*p* > 0.05) (Figure 4A,B).

#### 3.3.4 Ramsay sedation scores

**Figure 5:**
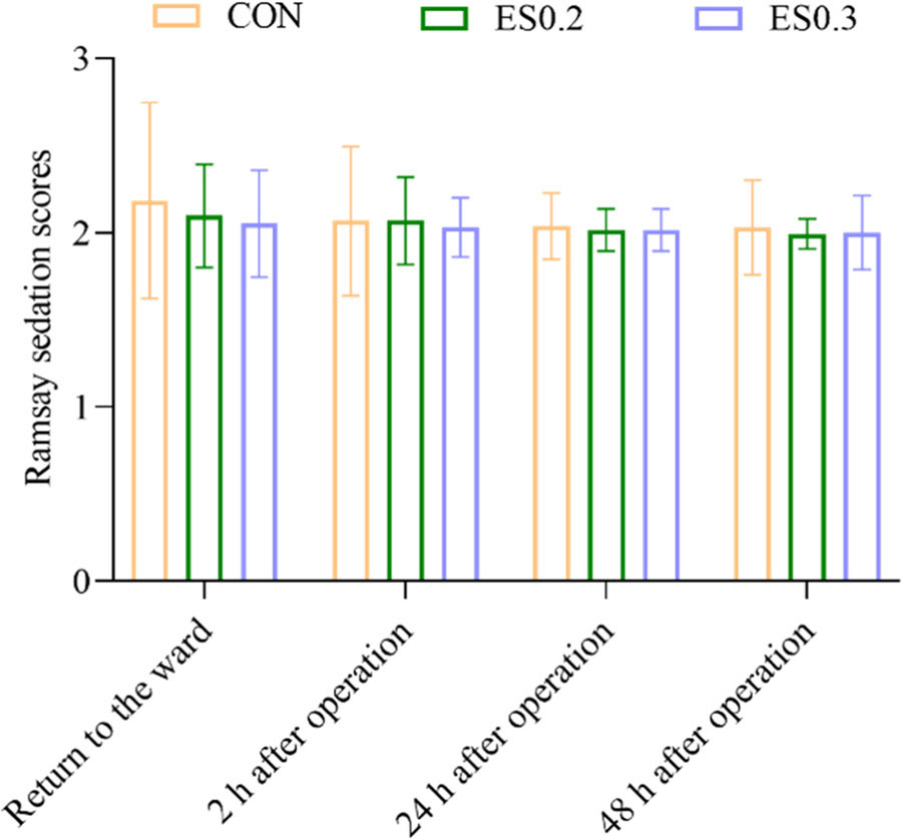
Ramsay sedation scores. The data are expressed as mean ± standard deviation. CON, placebo; ES0.2, 0.2 mg/kg group; ES0.3, 0.3 mg/kg group. [Color figure can be viewed at wileyonlinelibrary.com]

**Figure 6:**
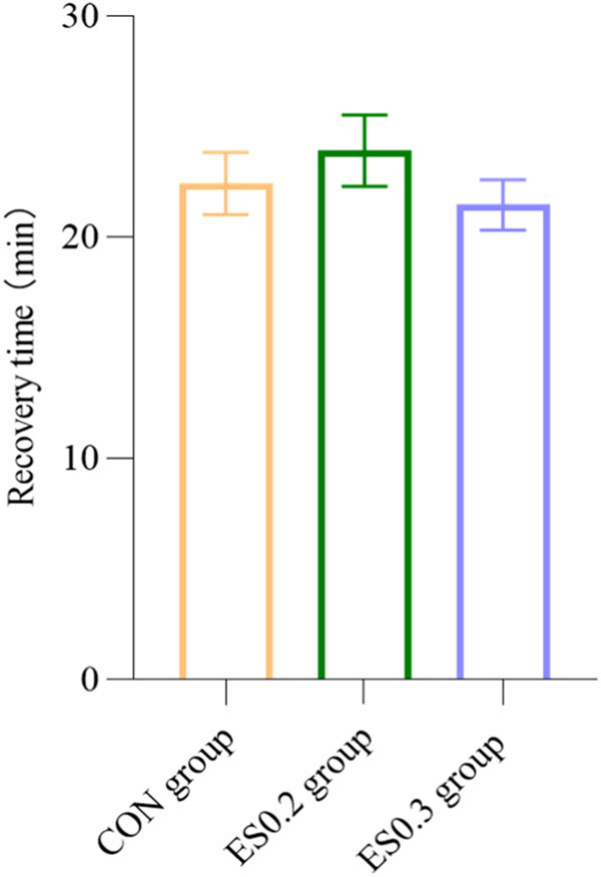
Recovery time. The data are expressed as mean ± standard error. CON, placebo; ES0.2, 0.2 mg/kg group; ES0.3, 0.3 mg/kg group. [Color figure can be viewed at wileyonlinelibrary.com]

There was no significant difference in postoperative Ramsay sedation scores at four‐time points (namely, the moment of returning to the ward, 2, 24, and 48 h after operation) among the three groups (*p* > 0.05) (Figure 5). There was no significant difference in recovery time between the three groups (*p* > 0.05) (Figure 6).

#### 3.3.5 Adverse events

##### Postoperative nausea and vomiting (PONV)

There was no significant difference in the incidence of PONV among the three groups within 2, 2–24, and 24–48 h (*p* > 0.05) (Table 5).

**Table 5:** Incidence of PONV.

	CON (*N* = 138)	ES0.2 (*N* = 138)	ES0.3 (*N* = 135)	*X* ^2^	*p* Value
0–2 h	13.8	15.9	12.6	0.652	0.722
2–24 h	32.6	22.5	34.8	2.742	0.254
24–48 h	3.6	4.3	3.0	0.373	0.830

*Note*: Data are presented as the percentage.

Abbreviations: CON, placebo; ES0.2, 0.2 mg/kg group; ES0.3, 0.3 mg/kg group.

##### Neurological adverse events

**Figure 7:**
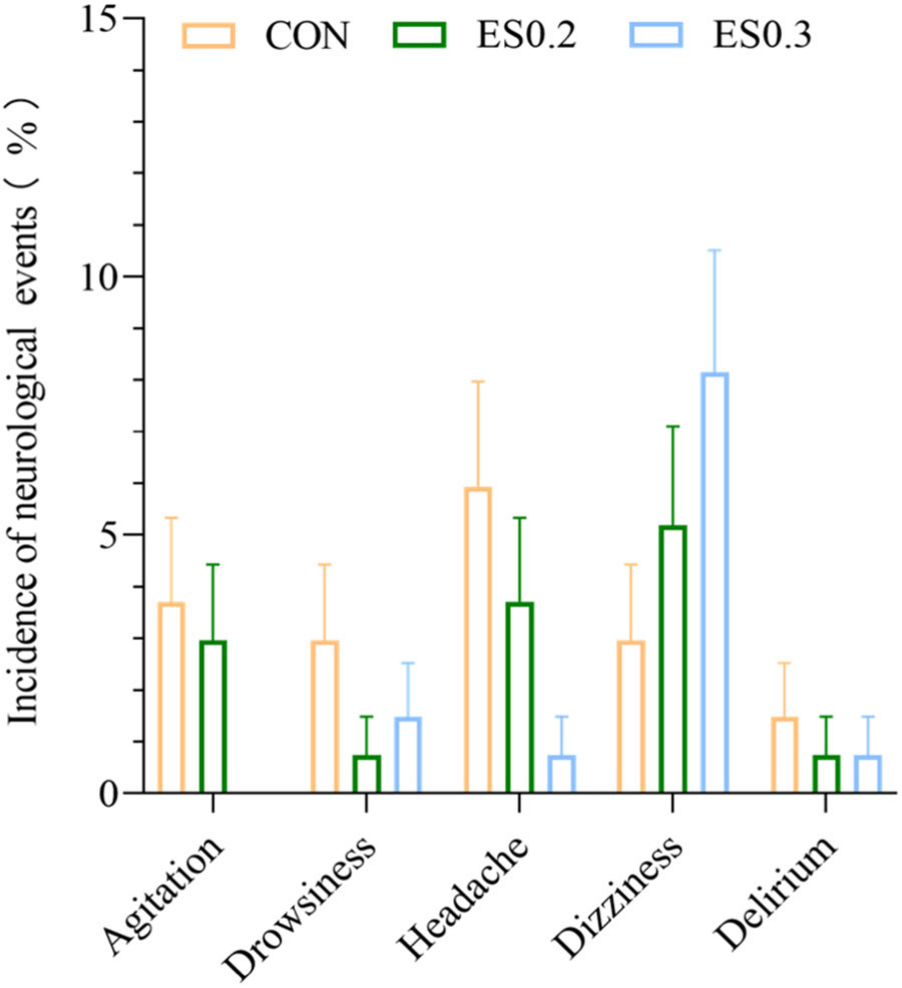
Incidence of neurological events. The data are expressed as mean + standard deviation. CON, placebo; ES0.2, 0.2 mg/kg group; ES0.3, 0.3 mg/kg group. [Color figure can be viewed at wileyonlinelibrary.com]

There was no significant difference in the incidence of neurological adverse events (agitation, drowsiness, headache, dizziness, and delirium) among the three groups (*p* > 0.05). The number of patients with dissociative anesthesia was 0 in the three groups (Figure 7).

#### 3.3.6 Anesthetic satisfaction

**Figure 8:**
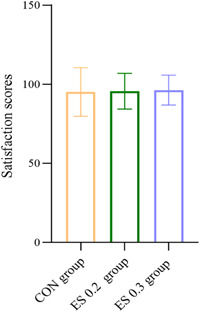
Satisfaction scores. The data are expressed as mean ± standard deviation. CON, placebo; ES0.2, 0.2 mg/kg group; ES0.3, 0.3 mg/kg group. [Color figure can be viewed at wileyonlinelibrary.com]

The total score of the assessment was 100 points. It consists of four data (whether there was fear of anesthesia, whether fear was eliminated after surgery, satisfaction with anesthesia, and satisfaction with postoperative analgesia), which accounts for 25 points each. The scores of the CON group, ES0.2 group, and ES0.3 group were 95.1, 95.6, and 96.3, respectively, with no significant difference (*p* > 0.05) (Figure 8).

## 4 DISCUSSION

Currently, S‐ketamine is mainly used for the non‐perioperative treatment of major depression and general perioperative anesthesia. As an antidepressant, it rapidly improves mood and symptoms and prevents attempts of suicide by directly targeting the glutamate system.^17^, ^18^ As a general intravenous anesthetic, it exerts a sedative effect while also exerting analgesic effects by acting on opioid receptors, NMDA receptors, and glycine receptors.^19^ Because of this property, S‐ketamine is favored by some anesthesiologists. And S‐ketamine has been reported to be effective in the prevention and treatment of neuropathic pain.^20^ In this trial, we found that subanesthetic‐dose S‐ketamine at 0.2–0.3 mg/kg in general anesthesia induction can reduce postoperative pain and opioid consumption without increasing the occurrence of anesthesia risk.

This study found that subanesthetic doses of S‐ketamine (0.2–0.3 mg/kg) at perioperative intervals effectively relieved postoperative pain. For both noninvasive and invasive procedures, we found a postoperative pain relief effect in two S‐ketamine groups according to the visual analogue scale first. At the same time, the analgesic pump compression results showed that the addition of S‐ketamine can reduce the occurrence of hypoanalgesia. And the 0.3 mg/kg dose group had a better analgesia effect and duration time. S‐ketamine has been shown to relieve postoperative pain in other studies in some way. Herman reported that applying low‐dose S‐ketamine (0.25 mg/kg) in patients undergoing elective major abdominal surgery can inhibit hyperalgesia and prolong analgesia time.^21^ To study acute and chronic pain in thoracoscopic pulmonary surgery, S‐ketamine (0.1 mg/kg) was administered after anesthesia induction and maintained at 0.1 mg/kg/h during the operation. Postoperative PICA (sufentanil 0.03 μg/kg/h + S‐ketamine 0.015 mg/kg/h) could effectively reduce acute and chronic pain after surgery.^22^ In this study, we found that the perioperative application of subanesthetic dose S‐ketamine at 0.2–0.3 mg/kg can effectively relieve postoperative pain and the 0.3 mg/kg dose of analgesia overwhelms the other two doses. Apparently, this is because the effect of the drug increases as the dose increases before the maximum dose is reached.

S‐ketamine at the dose of 0.3 mg/kg can reduce the postoperative opioids used, but such an effect could not be found with the dosage of 0.2 mg/kg. Opioids including nalbuphine, tramadol, dezocine, butorphanol, and sufentanil are used in this study for postoperative analgesia management. The efficacy intensities of the five remedial drugs are different and inconvenient to count, so we converted the doses into morphines according to the efficacy intensity relationship of these opioids. Nalbuphine is 0.8 times of morphine; tramadol is 0.1 times of morphine; dezocine is 1 times of morphine; butorphanol is 7 times of morphine; and sufentanil is 1000 times of morphine.^23^, ^24^, ^25^, ^26^ We finally found that 0.3 mg/kg S‐ketamine can exert the thrifty effect of opioids. Some studies have shown that using S‐ketamine can reduce opioid consumption. Brink observed patients taking S‐ketamine (0.25, 0.5, and 0.75 mg/kg) and oxycodone for self‐controlled analgesia after lumbar fusion. A significant reduction in cumulative 24 h opioid consumption was detected in the 0.75 mg/kg group.^27^ Infusion of 0.3 mg/kg/h S‐ketamine during gynecologic endoscopic surgery can reduce postoperative opioid consumption.^28^ Continuous intravenous infusion of subanesthetic esketamine at a rate of 0.25 mg/kg/h can significantly reduce postoperative opioid consumption and improve the patient's outcomes.^29^ Perioperative use of S‐ketamine can indeed reduce the use of opioids, but the dosage and methods used in the previous administration were different from those in this study. And the safety analysis of its use was lacking. Finally, based on the results of the two study dose groups in this study, we hypothesized that S‐ketamine exerts a dose‐dependent opioid thrifty effect. But it was necessary to pay attention to dose control in the clinic to avoid adverse reactions induced by large doses.

Induction of anesthesia with subanesthetic doses of S‐ketamine (0.2–3 mg/kg) does not unduly interfere with vital signs and postoperative sedation scores. S‐ketamine at 0.2 mg/kg does not cause significant hemodynamic changes. This is consistent with the results of the study on S‐ketamine combined with propofol in painless gastrointestinal endoscopy at a subanesthetic dose.^30^ However, the HR of the patients from the ES0.3 group increased but there was no significant increase in the incidence of tachycardia. Increased HR is correlated with the sympathomimetic effect of S‐ketamine.^14^ It could increase the content of circulating catecholamines, antagonize the sympathetic inhibition of other general anesthetics to a certain extent, reduce adverse fluctuations in HR, and even accelerate HR. The mean arterial pressure and oxygen saturation of the two S‐ketamine groups were stable. It explained that the two S‐ketamine groups in this study did not enhance the risk of intraoperative hypertension and tachycardia. In addition, this study found that S‐ketamine (0.3 mg/kg) significantly reduced the proportion of using the vasoactive drug in patients intraoperatively. It is consistent with a study on S‐ketamine in treating vascular response to depression. Intravenous injection of S‐ketamine (0.5 mg/kg) reduced HR and blood pressure fluctuations, thus stabilizing hemodynamics.^31^ This is because hemodynamic disorders in clinical anesthesia often manifest as negative changes caused by inhibition of the sympathetic system, but the sympathomimetic effect of S‐ketamine counteracts this unfavorable fluctuation. The intraoperative partial pressure of end‐tidal carbon dioxide and postoperative respiratory rate were stable in the three groups. Therefore, S‐ketamine 0.2–0.3 mg/kg does not provoke disturbances of vital signs.

Induction of anesthesia with subanesthetic doses of S‐ketamine (0.2 and 0.3 mg/kg) does not result in excessive sedation. In this study, we found that S‐ketamine, which acts as a sedative, does not interfere with patients' postoperative sedation scores or delayed recovery when administered during anesthesia induction. Meanwhile, there are few reports about S‐ketamine induction could increase the anesthetic recovery time.

Such a dose of S‐ketamine (0.2–0.3 mg/kg) does not increase the incidence of PONV. PONV effects of S‐ketamine are considered dose‐dependent like those triggered by ketamine. Studies have shown that combining ketamine at doses of 0.5–1 mg/kg anesthesia applied in pediatric magnetic resonance imaging and laparoscopic cholecystectomy in adults could increase the incidence of PONV.^32^, ^33^ Nevertheless, adjuvant fentanyl with ketamine at 0.3 mg/kg for self‐controlled intravenous analgesia after thoracic spine surgery did not increase the incidence of PONV.^34^ So, it could be thought that S‐ketamine at the dose of 0.2–0.3 mg/kg was not considered sufficient to increase the incidence of nausea and vomiting. Furthermore, someone has emphasized that low‐dose S‐ketamine did not increase the incidence of these side effects in endoscopic retrograde cholangiopancreatography (ERCP).^35^

Induction of anesthesia with subanesthetic doses of S‐ketamine (0.2–0.3 mg/kg) could be considered without causing dissociative anesthesia and increasing the incidence of other neurological side effects such as agitation, drowsiness, dizziness, and delirium. First, dissociative anesthesia refers to a state of stupor after injection of ketamine or S‐ketamine. The symptoms are loss of consciousness but eyes open staring, nystagmus, light reflex, cough reflex, swallowing reflex, increased muscle tension, and a few patients appear lockjaw and involuntary limb movement phenomenon.^36^ No cases of dissociative anesthesia occurred in the two dose groups in this study. Then, a study found that high doses of S‐ketamine might induce reversible neuropsychiatric side effects, such as delirium and dizziness; on the contrary, these side effects of low S‐ketamine doses are well tolerated, reversible, and of low incidence.^37^ This founding is consistent with ours.

Based on the assessment of the patient's vital signs, sedation, nervous system, and gastrointestinal adverse reactions, we found that subanesthetic‐dose S‐ketamine at 0.2–0.3 mg/kg could be used safely in general anesthesia induction.

Regarding patients' anesthesia satisfaction, there is an upward trend in the three groups. A study found that S‐ketamine nasal spray can improve the quality of anesthesia induction in pediatric strabismus surgery.^38^ And dexmedetomidine combinates S‐ketamine for induction of anesthesia in children could improve anesthesiologists' satisfaction. ^39^ As for patients' satisfaction, there is no clear difference but an uptrend with the dose of S‐ketamine increase among the three groups in this study. The satisfaction evaluation of this study mainly covers four aspects: whether there was fear of anesthesia before the operation; whether fear was eliminated after surgery; feelings after anesthetic emergence; and postoperative analgesia. The dissatisfaction factors were the patient's fear of anesthesia and the occurrence of nausea and vomiting. The latter was considered to be caused by opioids or intraoperative procedures of gastrointestinal surgery.

The major limitation of our study is that we need to include all possible dose study groups, which could be an interesting item for further research. And we do have not enough open surgery cases because the development of surgery has led to a sharp decline in the number of open operations.

In summary, subanesthetic‐dose S‐ketamine at 0.2–0.3 mg/kg in general anesthesia induction can safely and effectively reduce postoperative pain and save postoperative opioid consumption. In particular, S‐ketamine at a dose of 0.3 mg/kg has a better analgesic effect, stronger opioid thrifty effect, lower rate of vasoactive drug use, and a tendency to increase the heart rate without increasing the incidence of tachycardia. The use of subanesthetic‐dose S‐ketamine at 0.2–0.3 mg/kg as a perioperative intravenous general anesthesia is worthy of clinical promotion, and the dosage at 0.3 mg/kg has a prominent advantage.

## AUTHOR CONTRIBUTIONS

Jun‐Ding, Han‐Rui Qin, Man Luo, Xu Fang, Dan‐Dan Tan, Xue‐Feng Ren, Yong‐Guo Zhang, Tao Luo, and Lei Chen collected data. Yun‐Mei Yu wrote the original draft. Wan‐Qiu Yu edited the draft, and Zhao‐Qiong Zhu supervised the work. All authors have read and agreed to the published version of the manuscript.

## CONFLICT OF INTEREST STATEMENT

The authors declare no conflict of interest.

## ETHICS STATEMENT

The study was approved by the Ethics Committee of the Affiliated Hospital of Zunyi Medical University (approval number: KLL‐2020‐049) and registered at the Chinese Clinical Trial Registry (registration number: ChiCTR2100046703).

## Data Availability

The data supporting this study's findings are available on request from the corresponding author.
